# Formation and Characterization of Mycelium–Potato Protein Hybrid Materials for Application in Meat Analogs or Substitutes

**DOI:** 10.3390/foods13244109

**Published:** 2024-12-19

**Authors:** Ramdattu Santhapur, Disha Jayakumar, David Julian McClements

**Affiliations:** Department of Food Science, University of Massachusetts, Amherst, MA 01003, USA; rsanthapur@umass.edu (R.S.); djayakumar@umass.edu (D.J.)

**Keywords:** potato protein, mycelium, biopolymer composites, alternative proteins, hybrid products

## Abstract

There is increasing interest in the development of meat analogs due to growing concerns about the environmental, ethical, and health impacts of livestock production and consumption. Among non-meat protein sources, mycoproteins derived from fungal fermentation are emerging as promising meat alternatives because of their natural fibrous structure, high nutritional content, and low environmental impact. However, their poor gelling properties limit their application in creating meat analogs. This study investigated the potential of creating meat analogs by combining mycoprotein (MCP), a mycelium-based protein, with potato protein (PP), a plant-based protein, to create hybrid products with meat-like structures and textures. The PP-MCP composites were evaluated for their physicochemical, rheological, textural, and microstructural properties using electrophoresis, differential scanning calorimetry, dynamic shear rheology, texture profile analysis, confocal fluorescence microscopy, and scanning electron microscopy analyses. The PP-MCP hybrid gels were stronger and had more fibrous structures than simple PP gels, which was mainly attributed to the presence of hyphae fibers in mycelia. Dynamic shear rheology showed that the PP-MCP hybrids formed irreversible heat-set gels with a setting temperature of around 70 °C during heating, which was attributed to the unfolding and aggregation of the potato proteins. Confocal and electron microscopy analyses showed that the hybrid gels contained a network of mycelia fibers embedded within a potato protein matrix. The hardness of the PP-MCP composites could be increased by raising the potato protein content. These findings suggest that PP-MCP composites may be useful for the development of meat analogs with more meat-like structures and textures.

## 1. Introduction

Alternative protein sources are increasingly being used to formulate foods to mitigate the adverse environmental impacts associated with the production of animal proteins, especially meat [1]. At around 14–18% of the total, livestock production is a major contributor to global greenhouse gas emissions (GHGs). Deforestation for grazing pasture, fertilizer use for feed crops, and methane from ruminant digestion have been reported to be the main causes of GHGs in this sector [2,3,4]. Animal husbandry also utilizes considerable amounts of land and water, which exacerbates biodiversity loss and environmental degradation. Switching to alternative protein sources could greatly reduce these impacts, slowing climate change and advancing global sustainability [5,6]. Researchers have reported that substituting conventional meat products for plant-based, mycoprotein, or cultured meat products could reduce agricultural GHG emissions by over 50% by 2050 in high-consumption areas [7,8]. Alternative protein sources also have the potential to improve public health by reducing the risks of diet-related diseases that have been linked to excessive meat intake, such as cancer and cardiovascular disease [9,10]. Thus, alternative proteins have considerable potential for reaching global climate goals and promoting human health. Nevertheless, there are advantages and disadvantages associated with each individual kind of alternative protein source for formulating meat analogs and substitutes. For example, plant proteins are relatively abundant and affordable, but it is often challenging to accurately mimic the fibrous structure of real meat products using them, which reduces consumer acceptance [11,12]. In contrast, mycoproteins can better mimic the fibrous structure of meat products, but they are more difficult to produce affordably on a large scale and they cannot form strong gels when used alone [13]. Consequently, it is beneficial to create hybrid products that combine the benefits of these two alternative protein sources.

Mycoproteins are becoming popular for formulating meat analogs or substitutes because of their high protein content, positive health benefits, and low environmental impact [14]. This source of alternative proteins is usually produced in bioreactors by fungal fermentation, and then isolated and purified before being converted into meat substitutes [15,16]. Compared to animal proteins, mycoprotein has a far smaller carbon footprint and uses less energy and water. Indeed, the carbon footprint of mycoprotein is around ten times lower than that of beef and four times lower than that of chicken. A recent environmental and nutritional life cycle analysis of different protein-rich food sources found that global warming potential, water use, land use, and pollution were much less for mycoprotein than for beef, appreciably less than for chicken, and appreciably more than for plant proteins (tofu) [17]. The higher impact of mycoprotein-based products on the environment than plant protein-based ones has been attributed to the relatively high amounts of energy and raw materials required to run the bioreactors used to generate the mycelia [17]. Mycoprotein-based food products are rich in proteins, dietary fibers, vitamins, and minerals, and low in fat, which may lead to nutritional and health benefits [15]. Indeed, consumption of mycoproteins has been reported to stimulate the insulin response, promote digestive health, increase satiety, reduce cholesterol levels, and modulate blood sugar levels [15,16,18]. Consequently, mycelium-based food products have great potential to contribute to the world’s growing protein requirements [18].

Plant proteins are another important alternative protein source used to formulate meat analogs and substitutes [19,20,21]. Potato protein is a non-allergenic source of plant proteins [22]. Potatoes typically have a relatively low total protein content (1 to 2%). However, there are large quantities of protein-rich wastewater produced by the potato processing industry that can be converted into a value-added nutritional and functional ingredients for application in foods and other products [23]. The proteins isolated from potatoes are mainly comprised of patatin (35–40%) and protease inhibitors (30–40%) [22]. Potato proteins are considered to be a good nutritional source of proteins because they contain all the essential amino acids required to maintain human health and wellbeing [24,25]. Moreover, potato proteins can form strong heat-set gels because the protein molecules are typically in a native state in commercial ingredients [22,26,27], which is often not the case for more common commercial plant protein ingredients, such as those isolated from soybeans or peas [28,29].

In our previous study, we examined the possibility of creating hybrid products by combining mushrooms with either whey proteins [30] or potato proteins [31]. These studies showed that the introduction of the mushroom extracts into the protein gels created hybrid products that had a more fibrous structure than the original protein gels. However, the presence of the mushroom extracts could either increase or decrease the mechanical strength of the protein gels, depending on the system. Consequently, more research is needed to determine the factors that impact the structural and mechanical properties of hybrid products formed from different sources of alternative proteins. In the current study, we examined the properties of hybrid food products containing mycoproteins and potato proteins. The mycoproteins were selected because of their ability to create fibrous structures and their good nutritional profile, while the potato proteins were selected because of their ability to form firm heat-set gels and their high protein content. In particular, we examined how the appearance, rheology, texture, and microstructure of these hybrid products depended on their composition. It should be noted that other researchers have recently examined the impact of mineral ions on the properties of mycelium–potato protein hybrids formulated from a different kind of mycelium, i.e., *Fusarium venenatum* [32]. The results of this study suggested that electrostatic interactions played a key role in the formation and properties of these hybrids.

The information obtained in our study may be useful for creating more sustainable alternatives to meat products.

## 2. Materials and Methods

### 2.1. Materials

Mycelium was generously provided by the Better Meat Co. (Sacramento, CA, USA). Potato protein (“PP200,” Solanic^®^200) was kindly supplied by Royal Avebe (Veendam, The Netherlands). The company specified that the powdered potato protein ingredient contained 90.5% protein, 7.0% water, 2.9% salt, less than 0.2% carbohydrates, and less than 0.1 g fat (*w*/*w*). Sodium chloride (NaCl) was obtained from the Sigma-Aldrich Co. (St. Louis, MO, USA).

### 2.2. Mycoprotein Preparation

The mycoprotein samples were initially provided by the manufacturer in the form of dried pellets. Proximate analysis of these mycoprotein samples was carried out using standard AOAC methods, as described in our previous study on mushrooms [31]. The mycoprotein pellets were then converted into a fine powder using a food processor operated at 6000 rpm for 90 s. This powder was then sieved through a 20 mm mesh to achieve a uniform particle size. Moreover, sieving removed any large insoluble aggregates that might have sedimented during the preparation of the gels, thereby leading to inhomogeneous samples and inconsistent results. The powder was then hydrated in deionized water for 4 h before being blended into a paste using a meat blender. These samples were then utilized to prepare the hybrid products used for the rheological, textural, and microstructural tests.

### 2.3. Preparation of Mycoprotein–Potato Protein Hybrids

Potato protein (PP) and mycoprotein (MCP) powders were blended together in various ratios and then dispersed in double-distilled water. The mixtures were then stirred overnight at 350 rpm using a magnetic stirrer to ensure proper hydration and dispersion. A series of test samples was then prepared based on their ability to form heat-set gels: (i) *potato protein samples*: 5%, 10%, and 15% PP; (ii) *mycoprotein samples*: 15% MCP; and (iii) *hybrid samples*: 15% MCP with either 10% or 15% PP. Some dispersions were analyzed directly for zeta potential, differential scanning calorimetry, and dynamic shear rheology, while others were converted into heat-set gels by heating then in a water bath at 90 °C for 30 min and then used for texture profile and microscopy analyses. The PP and MCP concentrations used in this study were selected with the aim of creating final hybrid products with a protein level, texture, and structure somewhat similar to that found in real meat.

### 2.4. Zeta Potential Analysis

Microelectrophoresis (Zetasizer Nano ZS, Malvern Instruments, Malvern, Worcestershire, UK) was used to characterize the electrical properties of the potato protein, mycoprotein, and hybrid samples. This method is based on measuring the direction and speed of movement of molecules or particles within an oscillating applied electric field [33]. First, dispersions of 0.1% (*w*/*v*) of PP and/or MCP were prepared with pH values ranging from 3 to 8. The zeta-potential values of these dispersions were then measured using the microelectrophoresis instrument. The samples’ pH was adjusted to the required value using stock solutions of HCl and NaOH. At least four measurements were taken for every data point, and the average was calculated.

### 2.5. Differential Scanning Calorimetry

Differential scanning calorimetry (DSC) was used to identify and characterize any thermal transitions of the constituents in the different samples during heating. Protein and mycoprotein dispersions (50–70 mg) were put into high volume aluminum pans, precisely weighed, and then hermetically sealed to avoid evaporation losses during the measurement process. Following equilibration to 25 °C, the sample cells were heated from 25 to 100 °C at a rate of 3 °C/min using the DSC apparatus (DSC25, TA Instruments, New Castle, DE, USA), as previously mentioned [30]. The heat flow (W/g) versus temperature (°C) profile of the samples was recorded as they were heated.

### 2.6. Dynamic Shear Rheology Analysis

Initially, PP, MCP and/or hybrid dispersions (pH 7.0, 100 mM NaCl) were prepared by dispersing the powdered ingredients in double distilled water and then stirring overnight at 350 rpm at room temperature. The rheological properties of these different samples were then analyzed under a dynamic shear rheometer (HR20, TA Instruments, New Castle, DE, USA). Around 1.0 to 1.5 mL of the unheated test sample was placed on a parallel plate (40.00 mm, stainless steel, crosshatched, Peltier plate) sample cell. The upper plate was then lowered onto the sample. The edges of the sample were then covered with mineral oil and a solvent trap was placed on top of the sample to reduce any evaporation during heating [34]. All analyses were performed using a gap of 1000 μm between the parallel plates.

- Temperature ramp: The samples were initially held at 25 °C for five minutes after being placed on the measuring cell. The rheological tests were then performed while the samples were heated at a rate of 6 °C per minute from 25 to 90 °C. After the samples reached 90 °C, they were maintained there for 20 min before being cooled to 25 °C at 6 °C/min. The samples were then kept at this temperature for another ten minutes. For these experiments, a frequency of 1.0 Hz and a strain of 0.1% were employed.
- Frequency sweep: After the temperature sweep was completed, a frequency sweep was performed, which involved measuring the dynamic shear modulus at 25 °C as a function of oscillation frequency (0.1–100 rad/s). A relatively small strain amplitude (0.1%) was employed during these tests to be within the linear viscoelastic region.
- Strain sweep: After the frequency sweep was completed, a strain sweep was carried out on the samples, which involved increasing the strain from 0.01 to 1000% at a fixed temperature of 25 °C and frequency of 1 Hz.

### 2.7. Texture Profile Analysis

Initially, powdered PP and/or MCP were dispersed in double-distilled water and then stirred overnight at 350 rpm at room temperature (pH 7.0, 100 mM NaCl). These dispersions were then poured into a glass flask, which was then heated for 30 min at 90 °C in a water bath before being cooled for two hours in an ice tray. The resulting gelled samples were then removed from the glass flasks and cut into cubes (1 × 1 × 1 cm^3^). The textural characteristics of the gelled samples were then measured using an instrumental texture analyzer (TA-XT2, Stable Micro System, Surrey, UK) using single and double compression tests [35]:

- *Single compression test*: A single compression test was used to determine the Young’s modulus and fracture characteristics of the samples, which was performed using a cylindrical probe (P/50, 50 mm stainless cylinder). The operating parameters used were based on those reported in a previous study: final target strain of 90%, and pre-test, test, and post-test speeds of 2, 1, and 10 mm/s, respectively [30]. The resultant stress–strain profiles were used to compute the Young’s modulus, fracture stress, and fracture strain.
- *Double compression test*: A two-cycle compression/decompression program was used to ascertain the texture profile analysis (TPA) parameters of the samples. The following test parameters were used for these tests: final target strain was 50%, and pre-test, test, and post-test speeds were all set at 2 mm/s. The two different compression/decompression cycles were separated by 5 s. These experiments were also conducted using a cylindrical probe (P/50, 50 mm stainless cylinder) [30]. The generated force-distance profiles were used to calculate the samples’ hardness, resilience, cohesion, springiness, gumminess, and chewiness, which was carried out by the computer software. The meaning of these terms, as well as criticisms of their limitations, have been given elsewhere [36].

### 2.8. Microstructure Analysis

CLSM: A confocal laser scanning microscope (CLSM) equipped with a 10× eye piece lens and a 40× objective lens (Nikon d-Eclipse C1 80i, Nikon, Melville, NY, USA) was used to determine the microstructure of the hybrid gels. Gel samples were placed on a glass slide after being cut into thin slices (0.2 mm) using a sharp knife. The samples were then stained using dyes specific to polysaccharides, lipids, and proteins: calcofluor white (1 mg/mL in ethanol) for polysaccharides, Nile red (1 mg/mL in water) for lipids, and FITC (1 mg/mL in ethanol) for proteins [31]. After adding one or two drops of these dyes to the samples, the mixtures were incubated for one to two minutes. A paper tissue was then used to remove any extra dye without affecting the sample. After that, the samples were examined under a microscope and covered with a glass cover slip. The instrument software was used to gather, store, and analyze the digital images (NIS-Elements 4.2, Nikon, Melville, NY, USA).

SEM: The microstructures of all the samples were also analyzed using a scanning electron microscope (SEM, JCM-6000 NeoScope, JEOL, Tokyo, Japan). The samples were first cut into 5 mm3 cubes and then dehydrated using a freeze drier for 3 days [37]. Following that, gold was sputter-coated onto the samples (Cressington 108Auto; Redding, CA, USA). The microstructure of the samples was then investigated under low vacuum conditions using an accelerating voltage of 10 kV.

### 2.9. Statistical Analysis

Apart from the dynamic shear rheology measurements, which were carried out in duplicate, all the other experiments were carried out in triplicate using newly prepared independent samples. Microsoft Excel (version 2405 (Build 17628.20144) was used to compute the means and standard deviations of this data. R-program software (R version 4.3.1) was used to execute an ANOVA (post hoc Tukey HSD test) to identify significant differences between samples (*p* < 0.05). It was assumed that the experimental data followed a normal distribution around the mean for the statistical analysis.

## 3. Results and Discussions

### 3.1. MCP Proximate Analysis

The proximate analysis of the MCPs showed that they contained 35.3–50.4% protein, 2.86% fat, 6.48 moisture, and 5.78% ash. This relatively wide range of protein contents is given because mycoproteins contain appreciable amounts of chitin, which contains nitrogen. Consequently, the nitrogen content (8%) measured by the Dumas method is influenced by both the protein and the chitin content of the mycoprotein. For this reason, the protein content was calculated based on conversion factors reported in previous studies for fungal proteins, which ranged from 4.38 [38] to 6.25 [39].

### 3.2. Electrical Characteristics

**Figure 1:**
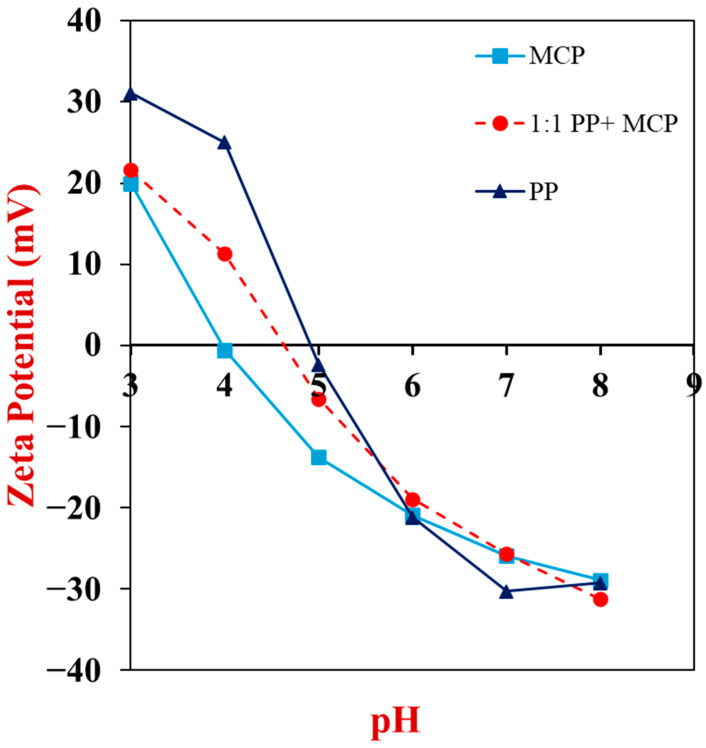
Impact of pH on the zeta potential of 0.1% (*w*/*v*) MCP, 0.1% (*w*/*v*) MCP + PP (1:1) and 0.1% (*w*/*v*) PP dispersions.

The pH of hybrid samples is important because it determines the electrical characteristics of the proteins and other components in the MCP and PP ingredients, which impacts their electrostatic interactions with each other. Previous studies have shown that electrostatic interactions play an important role in mycoprotein-plant protein hybrids [32,40]. For this reason, the zeta-potential values of the potato protein and mycoprotein dispersions were measured as a function of pH to provide some insights into their electrical characteristics (Figure 1). Potato proteins had an isoelectric point around pH 5, with the surface potential becoming increasingly negative at higher pH levels and increasingly positive at lower pH values. The change in zeta potential from positive, to zero, to negative as the pH was raised can be attributed to progressive conversion of the –NH_3_^+^ and –COOH groups into –NH_2_ and –COO^−^ groups, respectively [41]. Around the isoelectric point, the electrostatic repulsion between the potato protein molecules is expected to be relatively weak and therefore insufficient to prevent their aggregation.

The zeta-potential versus pH profile of the mycoproteins followed a similar trend to the potato proteins. The charge changed from highly positive at low pH to highly negative at high pH, with an isoelectric point around pH 4. This isoelectric point agrees with that reported for fungal proteins in other studies [42]. Mycoproteins are known to be assembled from biopolymers that can carry charge, such as proteins and chitin [43]. The pH-dependence of the zeta potential of the mycoprotein dispersions is consistent with the presence of proteins at their surfaces, however, the amino groups on chitin may also have made some contribution to their positive charge at low pH values. This effect might partly account for the fact that the mycoproteins had a lower isoelectric point than the potato proteins.

The change in zeta potential with pH for the mixed PP + MCP system followed a similar pattern to the pure PP and pure MCP systems. Indeed, for most pH values the zeta-potential of the mixed system was between than of the two pure systems. The isoelectric point of the mixed system was around pH 4.7, which was between that of the pure PP (pH 4.9) and pure MCP (pH 4.0) systems. At pH values between the isoelectric points of the potato protein and mycoprotein systems (i.e., pH 4.0 to 4.9), the PP and MCP have opposite charges, which could promote their aggregation through increased electrostatic attraction.

In the remainder of this study, we only prepared samples at pH 7, since this is close to the pH of most meat products. In principle, however, it may be possible to create different structures and textures by varying the pH of the hybrid products, but this was beyond the scope of the current work.

### 3.3. Appearance of the Samples

**Figure 2:**
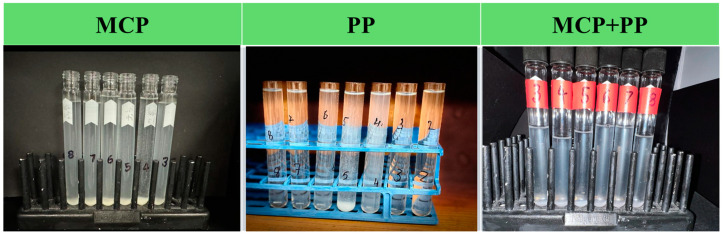
pH-dependence of the aggregation of the 0.1% (*w*/*v*) of MCP, 0.1% (*w*/*v*) PP and 0.1% (*w*/*v*) 1:1 MCP + PP samples.

Information about the pH-dependence of the aggregation of the different samples was obtained by taking digital photographs of them (Figure 2). The potato protein solutions were relatively clear at pH values far from their isoelectric point (pH 2, 3, 7, and 8), but turbid near their isoelectric point (especially pH 5), which is indicative of protein aggregation due to a reduction in electrostatic repulsion. The mycoprotein dispersions appeared turbid at all pH values, which can be attributed to light scattering by the small insoluble MCP fragments they contained. Moreover, a white precipitate formed at the bottom of these samples after a few hours of storage (Figure 2), which can be attributed to sedimentation of the MCP fragments caused by their relatively large size and higher density than water. The mixed MCP + PP dispersions contained precipitates at all pH values, which can mainly be attributed to the presence of the insoluble mycoprotein particles.

### 3.4. Differential Scanning Calorimetry

**Figure 3:**
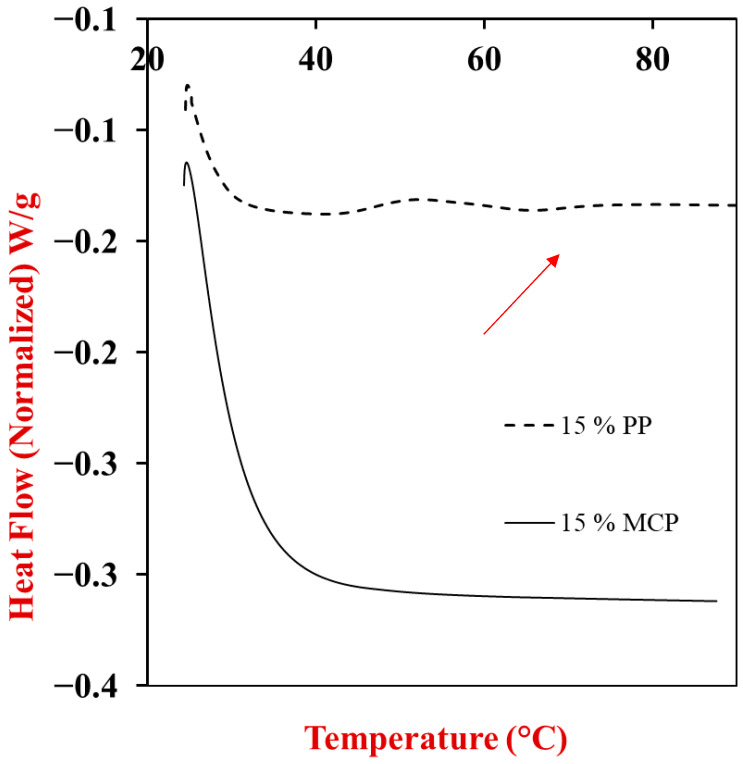
Differential scanning calorimetry profile of 15 wt% potato protein (PP) and 15 wt% mycoprotein (MCP) during heating from 25 to 100 °C with at 3 °C/min having denaturation temperature (T_d_) of 65.53 °C indicated with arrow.

The presence of any thermal transitions in the potato protein and mycoprotein dispersions was assessed using differential scanning calorimetry (DSC) analysis at pH 7. This pH is well above the isoelectric point of the potato proteins and mycoproteins, and so there should be a relatively strong electrostatic repulsion between them, which should reduce extensive protein aggregation [31]. The DSC thermograms for potato protein dispersions displayed a single, distinct endothermic peak upon heating, which is indicative of the thermal denaturation of the globular proteins [22,37]. The denaturation temperature (T_d_) of the potato proteins exhibited a slight dependence on concentration, with a T_d_ of 65.80 °C and 65.53 °C at PP concentrations of 10% and 15%, respectively (Figure 3). This result agrees with that reported in our previous studies for potato proteins [31]. The MCP dispersions did not exhibit any thermal transitions during heating, which may have been because the proteins had already been denatured during the manufacturing processes used to prepare the powdered mycoproteins. Moreover, some of the proteins in the MCP are embedded within cell walls containing chitin, which may increase their thermal stability [44,45].

### 3.5. Shear Rheology Analysis

Dynamic shear rheology was used to provide information about the impact of hybrid composition on their rheological properties.

#### 3.5.1. Temperature-Sweep Test

**Figure 4:**
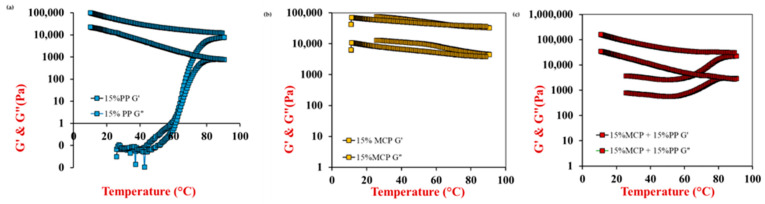
Temperature sweep results of (**a**) 15% potato protein (PP); (**b**) 15% mycoprotein (MCP); and (**c**) 15% MCP and 15% PP. The storage (G′) and loss (G″) moduli of the samples were measured as they were heated from 25 to 90 °C, held at 90 °C, and then cooled from 90 to 10 °C (strain = 0.1, frequency = 1 Hz). The red arrows show heating, while the blue arrows show cooling. For most temperatures, G′ > G″ for all samples, indicating they were predominantly elastic-like materials.

Initially, the temperature-dependence of the dynamic shear rheology of the potato protein and/or mycoprotein samples was determined. The storage modulus (G′) and loss modulus (G″) of these samples were measured as they were heated from 25 to 90 °C, held at this temperature, and then cooled to 25 °C. The rheology of 15% PP, 15% MCP, and 15% PP + 15% MCP hybrid samples are shown in Figure 4. Similar general trends were followed for samples containing 10% PP, 15% MCP, and 10% PP + 15% MCP but the shear modulus values were considerably lower. Notably, the samples containing 5% PP, 15% MCP or their hybrids did not form a gel or only formed a very weak gel that was easily disrupted.

For the samples containing 15% PP, the storage and loss moduli of the potato protein solutions were initially relatively low at 25 °C, which suggests that the proteins did not form a strong gel before heating (Figure 4a). This was probably because of the relatively strong electrostatic repulsion between the negatively charged protein molecules. When the temperature was raised, there was a slight decrease in the G′ and G″ values up to around 40 °C, which may have been due to dissociation of weak aggregates formed by the potato proteins at ambient temperature. Presumably, these aggregates formed due to the presence of some weak hydrophobic or hydrogen bonding between the protein molecules at lower temperatures. However, when the temperature was raised further, there was a modest increase in the shear moduli up to about 50 °C, followed by a steeper rise above about 60–70 °C, which can be ascribed to thermal denaturation and aggregation of the potato proteins leading to the formation of a 3D protein network with elastic-like properties [22]. Upon cooling from 90 to 25 °C, both G′ and G″ increased appreciably, indicating the formation of irreversible heat-set gels. The observed increase in gel strength during cooling can be attributed to strengthening of the hydrogen bonding within the protein matrix at lower temperatures [31].

For the 15% MCP sample, both G′ and G″ were initially relatively high at 25 °C, which was consistent with the observed paste-like properties of these samples. There was a slight decrease in shear moduli when the samples were heated and a slight increase when they were cooled (Figure 4b). However, there was not a dramatic change in the shear rheology of the samples during either cooling or heating, suggesting there were no distinct thermal transitions, which is consistent with the DSC measurements (Figure 3). Notably, G′ remained higher than G″ during heating and cooling, indicating that they were predominantly elastic-like materials. Nevertheless, they were very soft solids, which were unsuitable for creating meat substitutes or analogs on their own. The reasons that the mycoproteins could not form strong gels may have been because the proteins were already denatured prior to heating or because they were trapped inside mycelium fibers and so they could not form a 3D network [40,46,47].

The mycoproteins are rich in proteins, dietary fibers, and micronutrients, and they naturally have a fibrous structure. These attributes are important to create meat analogs with good nutritional properties. However, the poor gelling properties of the mycoproteins limits their application for this purpose. Consequently, we examined the possibility of increasing the gel strength of mycoprotein-rich samples by blending them with potato proteins. The hybrid samples (15% PP + 15% MCP) initially had relatively high G′ and G″ values at 25 °C, which can mainly be attributed to the presence of the mycoproteins (Figure 4c). During heating, there was an initial softening of the hybrid hydrogels, which can be attributed to weakening of the hydrogen bonding between molecules at higher temperatures. However, when they were heated above about 60–70 °C there was a steep rise in gel strength, which can be attributed to unfolding and aggregation of the potato proteins. The gel strength then increased when the samples were cooled from 90 to 25 °C, which can be associated with strengthening of the hydrogen bonds between the biopolymers at lower temperatures. Notably, the final gel strength was higher for the hybrid samples than for the pure potato protein samples, which indicated that the addition of the mycoproteins strengthened the gels. It is possible that the fibers in the mycoproteins acted as active fillers, which are known to increase the elastic modulus of composite gels [30]. It should be noted that the final shear modulus (G′) of the hybrid samples after heating and cooling was quite similar to that reported for real cooked chicken under similar conditions [34]. Consequently, the hybrid products may have textural attributes that can match those of real meat products.

#### 3.5.2. Frequency-Sweep Test

**Figure 5:**
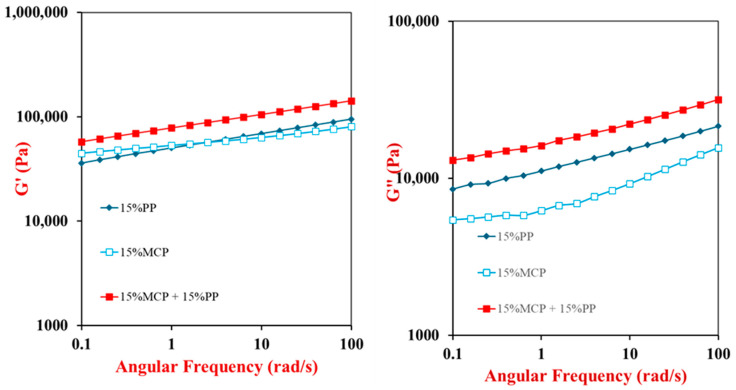
Frequency sweep results of 15% potato protein (PP), 15% mycoprotein (MCP), and 15% mycoprotein-15% potato protein (15% MCP + 15% PP) hybrid gels. The storage modulus (G′) and loss modulus (G″) of the samples were measured as the frequency was increased at a strain of 0.1% and 25 °C. For all frequencies, G′ > G″ for all samples, indicating they were predominantly elastic-like materials.

After the completion of the temperature-sweep test, a frequency-sweep test was performed to provide some insights into the dynamic properties of the hydrogels. For all the samples, there was a slight increase in the G′ and G″ values as the frequency was raised from 0.1 to 100 rad/s (Figure 5). This increase can be attributed to the fact that the biopolymers within the different hydrogels need a certain amount of time to rearrange themselves when an external shear stress is applied. At lower frequencies, the biopolymers have enough time to relax, but at higher frequencies, they do not, which leads to a more rigid and viscous material [37,48]. As expected, the magnitude of the shear modulus increased with increasing protein concentration in the PP samples. Moreover, the shear modulus of the hybrid samples was greater than that of the individual components with the corresponding PP concentration, which indicated that the presence of the mycoproteins again strengthened the potato protein gels. This effect may have been because some of the components within the MCP acted as active fillers that interacted with the surrounding protein network through attractive molecular interactions. For instance, MCP contains fibrous hyphae that are rich in β-glucan and chitin [43,49,50], which could form electrostatic, hydrophobic, and hydrogen bonds with the potato proteins [32].

Some insights into the nature of the hydrogels formed can be obtained by analyzing the dependence of the shear modulus on frequency [31,51]. The experimental data can be fit to a power law model:

log *G*′ = *z*′ log *ω* + *K*

Here, ω is the oscillation frequency, *z*′ is a constant related to the frequency-dependence of the shear modulus, and *K* is a constant related to the strength of the molecular interactions. The constant *z*′ represents the slope of a log-log plot of G′ versus ω. Gels can be classified into different types depending on the *z*′ value: covalent gels if *z*′ = 0; physical gels if *z*′ > 0; and viscous gels if *z*′ ≤ 1. The goodness of fit is evaluated from the coefficient of determination (R^2^).

The parameters obtained by fitting the power-law model to the experimental data are reported in Table 1. In our experiments, R^2^ was higher than 0.99 for all the samples, indicating a good fit of the model to the data. All our samples could be categorized as physical gels because the *z*′ values were > 0. The most likely origin of the physical crosslinks between the biopolymer molecules in the different samples is hydrophobic, hydrogen, and electrostatic bonding [32,52]. The *K* values (6.74 to 11.26) of the gels depended on their composition, which suggests that the overall strength of the molecular interactions in them depended on the types of biopolymers present. Hydrophobic interactions will have occurred between non-polar groups on the surfaces of the proteins when they were heated above their thermal denaturation temperature. Hydrogen bonding may have occurred between polar groups on the proteins and/or polysaccharides present, especially at lower temperatures. Electrostatic interactions may have occurred between cationic chitin and anionic protein molecules in the hybrid samples. The importance of these interactions has been highlighted in other studies on mycelium–protein interactions. For example, researchers have used computer simulations, microscopy, rheology, and spectroscopy to provide insights into the nature of the molecular interactions involved in the creation of hybrid gels from egg white proteins and mycoproteins [45]. They reported that hydrogen bonding, electrostatic interactions, and hydrophobic interactions all played an important role. In another study, researchers used microscopy, rheology, and electrophoresis to provide insights into egg protein-mycoprotein interactions [40]. They concluded that electrostatic interactions played a dominant role, but that hydrogen bonding and hydrophobic interactions were also likely to be important. Similar findings have also been reported by the same research group for mycoprotein-potato protein interactions [32]. Taken together, the results of these studies support our hypothesis about the role of electrostatic, hydrophobic, and hydrogen bonding interactions in determining the properties of the hybrid gels.

#### 3.5.3. Strain-Sweep Test

**Figure 6:**
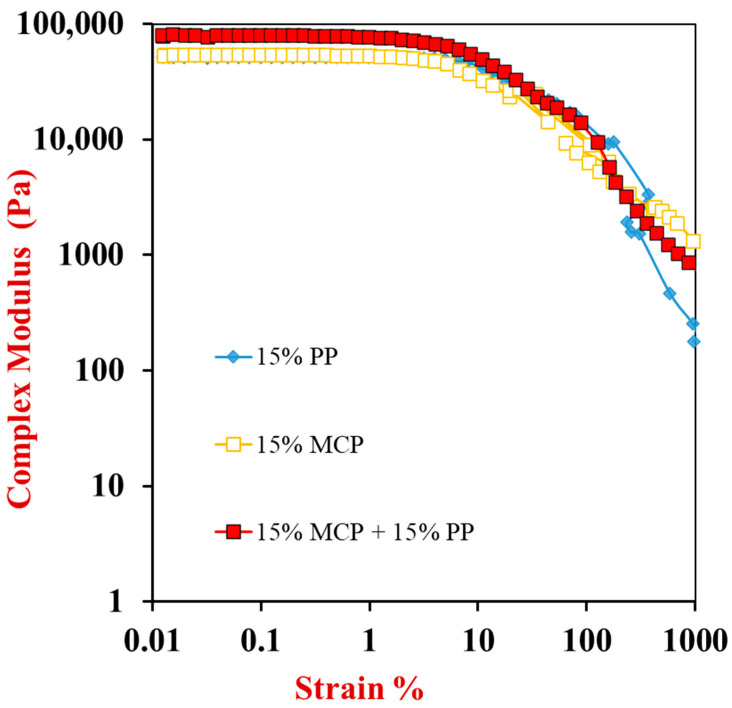
Strain sweep results of 15% pure potato protein,15% mycoprotein and15% potato protein-mycoprotein hybrid gels. The complex shear modulus (G*) of the samples were measured as the strain was increased from 0.01% to 1000% at 25 °C.

After the completion of the frequency-sweep test, a strain-sweep test was carried out to provide some insights into their non-linear properties at high deformations. The viscoelastic properties of the different gels were assessed by measuring their complex shear modulus (G*) when the strain was raised from 0.01% to 1000% (Figure 6).

For all samples, the shear modulus remained relatively constant when the strain was increased up to about 2–4% but then it decreased steeply. This indicates that the different gels had reversible elastic properties at relatively low deformations, where the applied stress was proportional to the resulting strain. This low deformation region is referred to as the “linear viscoelastic regime”. Above this region, the gel network is disrupted, and some yielding and flow may occur, which results in a decrease in the complex shear modulus. The strain sweep results therefore suggest that all the samples exhibited fairly similar breakdown profiles under shear. At higher strains, the applied shear stress was strong enough to disrupt the bonds between the different structural elements in the gel networks causing them to yield and flow.

### 3.6. Texture Profile Analysis

Further insights into the behavior of the different hydrogels when exposed to large deformations (like those experienced during mastication) were obtained using uniaxial compression testing [31]. For these tests, all the samples were heated to 90 °C for 30 min and then cooled to ambient temperature prior to analysis.

#### 3.6.1. Single Compression Tests

**Figure 7:**
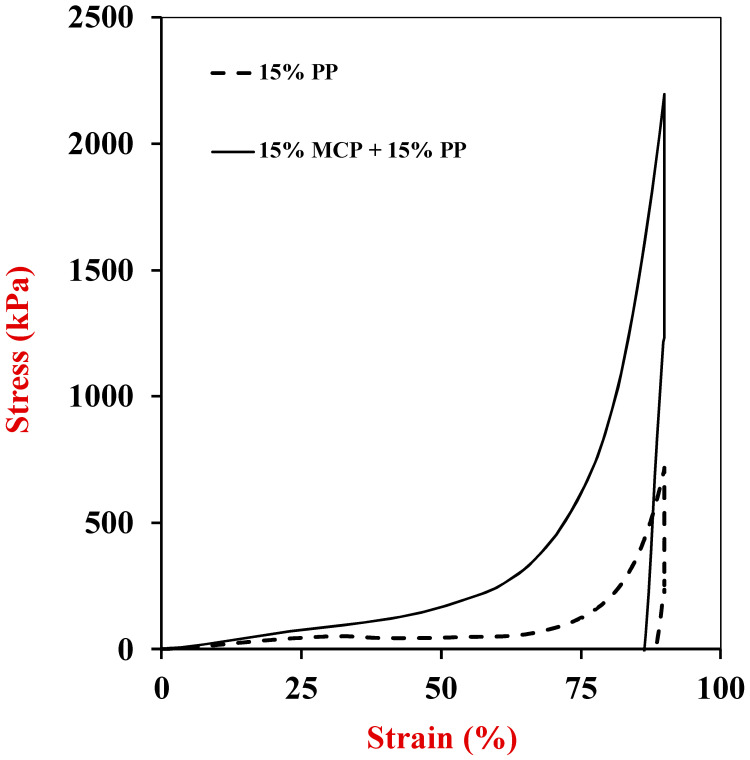
Effect of single composition on the stress–strain relationship of Potato protein and potato protein-mycelium hybrids during single compression-decompression experiments (25 °C).

A single compression test was used to measure the strain versus strain curves of the samples when they were compressed to a final strain of 90% (Figure 7). The Young’s modulus and fracture properties of the gels were then determined (Table 2). The Young’s modulus was calculated from the initial slope of the curves, whereas the breaking stress and breaking strain were determined from the first point when there was a break in these curves [35]. All the samples exhibited an initial rise in force with increasing strain, which indicated that they had some elastic-like properties. After reaching a certain strain threshold, all the samples then exhibited a notable increase in stress before ultimately failing, indicating their maximum loading capacity had been exceeded. The appreciable increase in force may have been because the surface area of the samples increased as they were compressed, and so they gave a greater resistance to compression. The samples may have broken at higher strains because the applied stresses exceeded the forces holding the different structures in the gel networks together. The breaking stress and strain values determined from the first observed break in the force-strain curves are reported in Table 2.

Consistent with the shear modulus experiments, the Young’s modulus of the 15% MCP + 15% PP hybrid gels was the highest. In addition, they also had the highest breaking stress (2143.5 kPa) and breaking strain (≥90.0%), which may be valuable for the formulation of meat analogs and substitutes. The enhanced mechanical properties of the hybrid gels may be attributed to a synergistic interaction between the MCP and PP components, which allows for a stronger and more robust biopolymer network to be formed. Indeed, fibrous fillers have been reported to be highly effective at increasing the mechanical strength of composite materials [53,54]. Notably, the Young’s modulus of the hybrid samples (15% MCP + 15% PP) analyzed in our study had fairly similar values to that of cooked chicken reported in a previous study [34], which may be useful for their commercial application as meat substitutes.

Overall, the incorporation of the MCP into the PP gels enhanced their strength and flexibility, which may be partly due to the fibrous structures in the mycoproteins.

#### 3.6.2. Double Compression Test

**Figure 8:**
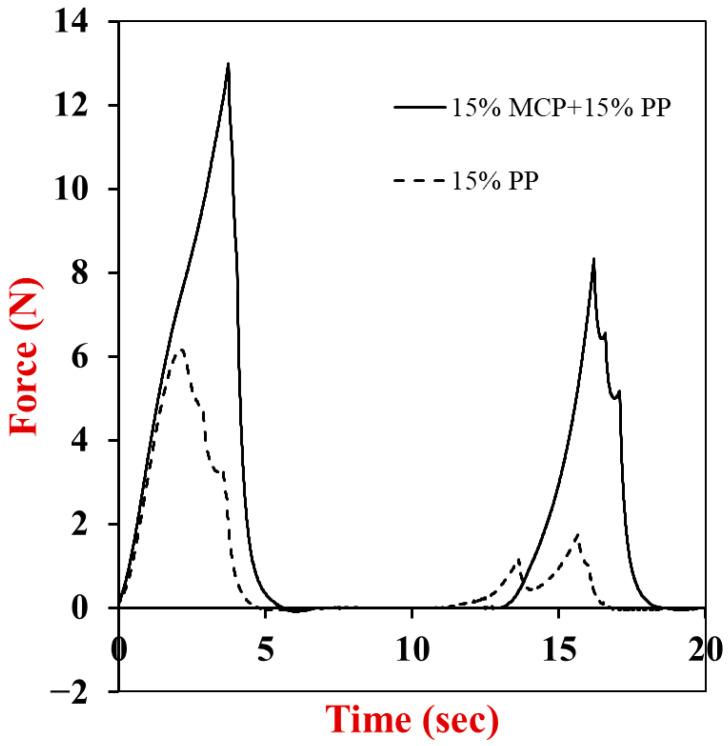
Double compression curves of 15% potato protein (15% PP) and 15% mycoprotein + 15% potato protein (15% MCP + 15% PP) hybrid gels. The force versus time curves were measured at 25 °C with 50% final strain and 2 mm/s pre-test, test, and post-test speeds.

A double compression test was then used to measure the texture profile analysis (TPA) parameters of the different gels (Table 3). Representative force *versus* time profiles for different samples are shown in Figure 8, which show that incorporating the MCP into the potato protein gels increased their resistance to deformation. For the pure potato protein gels and the hybrid gels, the hardness and chewiness increased appreciably as the PP concentration was raised from 5 to 15% (Table 3). This effect can be attributed to the presence of a stronger protein network at higher protein concentrations due to greater crosslinking of the potato protein molecules. The increase in hardness with PP concentration is consistent with the increase in shear modulus and Young’s modulus discussed earlier. The increase in chewiness with increasing potato protein content may be particularly important for the formulation of meat analogs and substitutes, as real meat products are usually characterized by a high degree of chewiness. There was not an appreciable dependence of the resilience (6.7–7.9%) or cohesiveness (0.23–0.33) of the pure potato protein samples on protein content. The resilience is a measure of how much the material regains its original shape and size after being compressed, while the cohesiveness is a measure of how well it can withstand a second deformation [36]. Our results suggest that all the pure potato protein samples exhibited relatively poor resilience and cohesiveness, which can be attributed to irreversible disruption of the 3D gel network formed by the proteins during compression.

The hardness and chewiness of the hybrid gels was greater than that of the equivalent potato protein gels. For example, the hardness of the 15% MCP + 15% PP gel was 16.4 ± 1.7 kPa, whereas that of the 15% PP gel was only 6.2 ± 0.3 kPa. Thus, the addition of the MCP increased the strength of the potato protein gels by nearly 165%, even though the MCP alone could not form a strong gel. As discussed earlier, this effect may have been because the MCP formed an interpenetrating network with the PP, or because some of the components in the MCP reinforced the molecular interactions between the potato protein molecules. There was not a strong dependence of the resilience (12.5–14.9%) or cohesiveness (0.51–0.59) of the hybrid gels on protein content. However, the resilience and cohesiveness values of the hybrid gels were significantly higher than those of the pure potato protein gels, which suggests that the presence of the mycoproteins somewhat increased their resistance to irreversible deformation during compression. This may have been because the fibrous hyphae in the MCP penetrated through the potato protein network, thereby holding it together better. The 5% PP and 15% MCP samples could not be analyzed using this method because they were too soft and runny.

### 3.7. Microstructure Analysis

Finally, the microstructures of the different heat-set gels were analyzed using scanning electron microscopy (SEM) and confocal laser scanning microscopy (CLSM) with fluorescence staining to provide some insights into the impact of the incorporation of the mycelium on the structure of the potato protein gels. It is often important to have a fibrous structure in meat analogs and substitutes. We hypothesized that the incorporation of the fibrous MCP into the PP gels would introduce this kind of structure in the hybrid gels. In addition, digital photographs of the different samples were taken to provide insights into the impact of their composition on their appearance. Only the 15% PP, 15% MCP, and 15% MCP + 15% PP hybrid gels were selected for the microstructure analysis because they had the strongest mechanical strength and were therefore the most suitable as meat substitutes and analogs.

#### 3.7.1. CLSM Analysis

**Figure 9:**
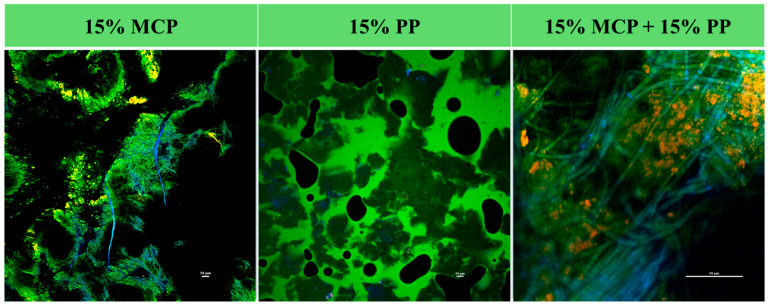
Confocal microscopy images of 15% mycoprotein (15% MCP), 15% potato protein (15% PP) and 15% mycoprotein + 15% potato protein (15% MCP + 15% PP) hybrid gels (25 °C). The images of the pure mycoprotein samples show they contained fibrous structures (stained blue and green), which were presumably chitin- and protein-rich hyphae. The images of the pure potato proteins showed that they contained large protein aggregates (stained dark green) dispersed in a protein-rich aqueous phase (stained light green). The black regions were probably holes formed during sample preparation. The images of the hybrid samples showed that they contained some fibrous structures (stained blue and green), which were presumably chitin- and protein-rich hyphae, distributed in a protein-rich network (stained green).

Confocal laser scanning microscopy with fluorescence staining was used to provide information about the microstructure of the different samples (Figure 9). In these images, the proteins were stained green, the polysaccharides blue, and the lipids red. The 15% mycoprotein gels had a complex interconnected network structure that consisted of fibrous proteins (green) and polysaccharides (blue). These fibers were probably fungal hyphae that consisted of chitin-rich walls with proteins packed inside [44]. There were some small yellowish-red regions in these images, which suggests that there were some lipids present, which is consistent with the proximate analysis of these samples (Table 1). There appeared to be many large pores (black regions) within these gels, which could affect the water-holding capacity, resilience, and texture of gels that include them. The fibrous structures found in the mycelia may be suitable for simulating those found in traditional meat products.

The 15% PP gels contained large dense irregular-shaped protein aggregates (dark green) dispersed within a dilute protein solution (light green). The large black holes seen in these images were voids in the gels that were introduced when they were cut into thin slices for microscopy analysis. These results are consistent with previous studies made on potato protein gels [52].

The CLSM images of the 15% mycoprotein and 15% potato protein hybrid gels showed that they had a highly heterogeneous network structure, with distinct regions corresponding to proteins (green), lipids (red), and polysaccharides (blue) (Figure 9). There appeared to be bundles of fibers from the mycelia wrapped around large protein-rich aggregates from the potato proteins. The complex microstructure of the hybrid gels would be expected to impact their functionality, including their water-holding capacity, resilience, and textural attributes [55].

#### 3.7.2. SEM Analysis

**Figure 10:**
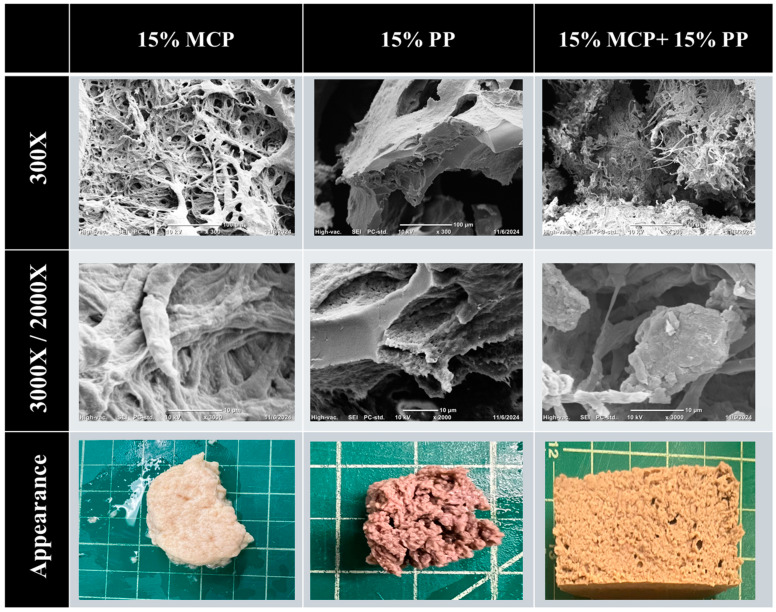
The scanning electron microscopy images of 15% mycoprotein, 15% potato protein and 15% mycoprotein + 15% potato protein hybrid gel. Scale bars are 100 μm for 300× and 10 μm for 3000× and 2000× for 15% potato protein. The digital photographs show the overall appearance of the samples before freeze drying.

Prior to SEM analysis, the gelled samples were freeze-dried at −80 °C to eliminate any moisture, which would interfere with the scanning electron microscopy measurements. Both low (300× magnification) and high (2000× magnification for 15% potato protein and 3000× for 15% mycoprotein and 15% mycoprotein + 15% potato protein hybrid gels) resolution images were taken of each of the samples to show their different levels of microstructure (Figure 10).

The low-resolution images of the 15% PP samples showed they consisted of a relatively smooth matrix with some large irregular pores, which were probably air bubbles embedded within a uniform potato protein network. The high-resolution images of these samples indicated that the pores had rough surface textures, which were probably clusters of protein aggregates. These microstructural features are likely to play an important role in determining the mechanical strength and gelling behavior of the potato proteins.

The low-resolution images of the 15% MCP samples showed that they had a highly porous fibrous structure, but that the fibers did not seem to be aligned in any particular direction. This was probably because the original mycoprotein samples were ground into a find powder prior to use, which destroyed some of their original fibrous structures. The high-resolution images of these samples indicated that they contained an intricate network of fibrous structures. Overall, these images show that the mycoproteins have a highly fibrous microstructure, which may be useful for creating meat analogs or substitutes with meat-like textures.

The SEM images of the 15% MCP + 15% PP hybrid gels indicated that they had microstructural features that combined those seen in the MCP and PP samples. The low-resolution images show there were some regions that were relatively smooth, which may have been the potato protein network, as well as some regions that were fibrous, which may have been the mycoproteins.

The digital photographs of the samples showed that they had different appearances depending on their compositions. The 15% MCP samples were a whitish color and had a highly heterogeneous fibrous surface texture. The 15% PP samples had a beige color and a “chunky” appearance. The hybrid samples had an appearance that had some features of the two separate components, with a light yellowish-brown color and a fibrous porous texture.

In summary, the hybrid gels appeared to contain mycoprotein fibers embedded in a potato protein network. The mycoprotein provided a fibrous structure, whereas the potato proteins provided mechanical strength. These hybrids may therefore have some of the attributes required to create solid-like fibrous meat analogs and substitutes. However, sensory studies on meat analogs or substitutes created from these hybrid materials would be needed to confirm this.

## 4. Conclusions

In conclusion, this study showed that hybrid gels could be assembled by combining mycoproteins and potato proteins together. Each of these alternative protein sources brings different attributes to the hybrids. The potato proteins form a relatively strong gel network due to their ability to unfold and aggregate with each other during heating, leading to the formation of an irreversible heat-set gel. In contrast, the mycoproteins cannot form strong gels during heating, but they can provide a desirable fibrous structure, as well as valuable nutrients, such as proteins, dietary fibers, vitamins, and minerals. Consequently, these hybrid gels may be useful for formulating meat analogs and substitutes. The mechanical properties of these hybrid gels could be modulated by altering their protein concentration, with the gel strength increasing within potato protein concentration. The mechanical strength of the hybrid gels was greater than that of the equivalent potato protein gels, which suggested that the presence of the mycoproteins strengthened the gel network, which may have been because the mycelia fibers behaved as active fillers in the potato protein network.

The results of this study may be useful for the design and production of a new generation of plant protein-mycoprotein hybrids as sustainable food sources. However, further research is required to fully understand the molecular interactions driving their rheological and structural properties and to optimize their formulations for broader applications. In particular, it will be necessary to include other ingredients in commercial hybrid products, such as lipids, flavors, colors, and preservatives to make them more desirable to consumers. In addition, it will be important to examine the impact of other kinds of alternative protein sources, such as those from other plants, insects, or microbial fermentation, on the formation and properties of hybrid products formulated with mycoproteins. Moreover, it will be important to carry out sensory tests on these products to determine if they are acceptable to consumers. In principle, the fibrous structures and good nutritional profile provided by mycelia should reduce the number of ingredients and amount of processing steps required to formulate meat analogs, thereby increasing their consumer acceptance. Indeed, a recent study of the factors affecting consumer acceptance of fungal proteins in meat substitutes found that the sensory attributes of the final products were the most important factor, but that the number of ingredients and degree of processing were also important [56]. This study also found that many consumers were unfamiliar with fungal proteins and had a negative opinion of them because they associated them with mold. Another recent consumer study of the factors impacting the adoption of fungi-based foods reported that several factors were important, including sensory characteristics, environmental benefits, nutritional effects, production practices and ingredients. There is also a need to carry out more detailed life cycle analysis studies on hybrid products formed from mycelia, to establish their potential environmental impacts compared to other protein sources [17]. Moreover, there is a need to assess the economic viability of these products in terms of ingredient costs and processing methods. Consequently, further efforts are required to create higher quality hybrid foods using minimal processing methods, and on educating consumers about the potential health and environmental benefits of these sustainable food sources.

## Figures and Tables

**Table 1:** Impact of composition on the parameters determined by fitting a power-law model to the experimental shear modulus versus frequency data for single and hybrid gels.

	Storage Modulus			Loss Modulus		
Sample	R^2^	Z′	K′	R^2^	Z″	K″
5% PP	0.999	0.112	6.75	0.995	0.114	5.04
10% PP	0.999	0.147	8.73	0.997	0.137	7.84
15% PP	0.999	0.139	10.82	0.997	0.134	9.33
15% MCP + 5% PP	0.994	0.100	10.00	0.951	0.158	8.01
15% MCP + 10% PP	0.999	0.117	10.42	0.988	0.139	8.75
15% MCP + 15 PP	0.999	0.130	11.27	0.990	0.129	9.73
15% MCP	0.989	0.081	10.81	0.940	0.168	8.70

**Table 2:** Impact of composition on Young’s modulus, breaking stress, and breaking strain of single and hybrid gels measured using a single compression test. The 5% PP and 15% MCP samples were not analyzed (NA) because they were too soft and runny.

Sample	Young’s Modulus (kPa)	Breaking Stress (kPa)	Strain (%)
5% PP	**-**	**-**	**-**
10% PP	0.520 ± 0.067	275 ± 16	≥90
15% PP	1.99 ± 0.23	567 ± 450	70 ± 35
15% MCP + 5% PP	0.141 ± 0.002	799 ± 73	≥90
15% MCP + 10% PP	0.323 ± 0.030	908 ± 28	≥90
15% MCP + 15% PP	2.71 ± 0.49	2144 ± 73	≥90
15% MCP	NA	NA	NA

**Table 3:** Impact of composition on the TPA parameters determined using a double compression test. The 5% PP and 15% MCP samples were not analyzed (NA) because they were too soft and runny.

Sample	Hardness (N)	Adhesiveness	Resilience (%)	Cohesion	Springiness (%)	Chewiness
5% PP	NA	NA	NA	NA	NA	NA
10% PP	1.7 ± 0.1	−0.18 ± 0.13	6.68 ± 0.98	0.33 ± 0.02	92.4 ± 4.9	0.51 ± 0.10
15% PP	6.2 ± 0.3	−0.12 ± 0.06	7.85 ± 0.53	0.23 ± 0.03	90.4 ± 4.3	1.26 ± 0.10
15% MCP + 5% PP	1.8 ± 0.4	−0.05 ± 0.03	12.46 ± 1.00	0.52 ± 0.03	83.2 ± 4.4	0.77 ± 0.21
15% MCP + 10% PP	4.4 ± 0.4	−0.03 ± 0.00	14.92 ± 0.22	0.59 ± 0.00	90.5 ± 1.4	2.35 ± 0.12
15% MCP + 15% PP	16.4 ± 1.7	−0.08 ± 0.02	13.05 ± 0.57	0.51 ± 0.01	84.1 ± 1.6	7.07 ± 0.71
15% MCP	NA	NA	NA	NA	NA	NA

## Data Availability

The original contributions presented in this study are included in the article. Further inquiries can be directed to the corresponding author.
